# ALS Pathogenesis and Therapeutic Approaches: The Role of Mesenchymal Stem Cells and Extracellular Vesicles

**DOI:** 10.3389/fncel.2017.00080

**Published:** 2017-03-21

**Authors:** Roberta Bonafede, Raffaella Mariotti

**Affiliations:** Department of Neuroscience, Biomedicine and Movement Sciences, University of VeronaVerona, Italy

**Keywords:** amyotrophic lateral sclerosis, ALS therapeutic applications, mesenchymal stem cells, extracellular vesicles, exosomes

## Abstract

Amyotrophic lateral sclerosis (ALS) is a fatal neurodegenerative disease characterized by progressive muscle paralysis determined by the degeneration of motoneurons in the motor cortex brainstem and spinal cord. The ALS pathogenetic mechanisms are still unclear, despite the wealth of studies demonstrating the involvement of several altered signaling pathways, such as mitochondrial dysfunction, glutamate excitotoxicity, oxidative stress and neuroinflammation. To date, the proposed therapeutic strategies are targeted to one or a few of these alterations, resulting in only a minimal effect on disease course and survival of ALS patients. The involvement of different mechanisms in ALS pathogenesis underlines the need for a therapeutic approach targeted to multiple aspects. Mesenchymal stem cells (MSC) can support motoneurons and surrounding cells, reduce inflammation, stimulate tissue regeneration and release growth factors. On this basis, MSC have been proposed as promising candidates to treat ALS. However, due to the drawbacks of cell therapy, the possible therapeutic use of extracellular vesicles (EVs) released by stem cells is raising increasing interest. The present review summarizes the main pathological mechanisms involved in ALS and the related therapeutic approaches proposed to date, focusing on MSC therapy and their preclinical and clinical applications. Moreover, the nature and characteristics of EVs and their role in recapitulating the effect of stem cells are discussed, elucidating how and why these vesicles could provide novel opportunities for ALS treatment.

## Introduction

Amyotrophic lateral sclerosis (ALS) is a fatal adult-onset neurodegenerative disease, first described by the neurobiologist Jean-Martin Charcot in the 1870s and which became initially known as Charcot’s sclerosis. In the USA, the disease is also known as Lou Gehrig disease, in honor of the baseball player who developed the disease in the 1930s. ALS has an incidence of 2–3/100,000 and a prevalence of 6–7/100,000 in Europe (Costa and de Carvalho, 2016), making it the most common motoneuron disease in human adults (Cleveland and Rothstein, 2001). In 90%–95% of cases there is no apparent genetic link (sporadic ALS, sALS), while the remaining 5%–10% of cases have a family history (familial ALS, fALS).

In general, the first symptoms appear at the mean age of 50 years for fALS, and 60 years for sALS, even if the onset can occur in very young individuals or in elderly people (Costa and de Carvalho, 2016). The disease is more common in males than in females, with an incidence of 1.5:1 (Zarei et al., 2015), although the incidence in men and women is about the same in fALS. The disease is fatal within 2–5 years after clinical onset; about 50% of patients die within 30 months from symptom onset, while about 10% of patients may survive for more than a decade (Forsgren et al., 1983; del Aguila et al., 2003).

Both sALS and fALS are characterized by selective degeneration of both upper motoneurons in the primary motor cortex, and lower motoneurons in the brainstem and spinal cord. However, some groups of motoneurons, including those that control pelvic muscles (Onuf’s nucleus in the sacral spinal cord) and eye movements (oculomotoneurons), are spared by the pathology. The reasons of this differential motoneuron vulnerability remain unknown (Comley et al., 2015).

The familial and sporadic forms of the disease appear clinically indistinguishable. Although the motoneuron degeneration is the main hallmark of the disease, ALS can be classified according to the district of symptom onset. Limb onset is the most common presentation of ALS, with progressive muscle weakness and wasting, body weight loss, fasciculations, emotional lability and cognitive dysfunction. In the bulbar onset, representing approximately 30% of ALS cases, the disease starts with difficulties in speech and swallowing (dysarthria and dysphagia), followed by limb symptoms (Pratt et al., 2012).

In sALS, the occurrence of the disease could be also due to a gene-environment interaction; sALS has been associated with “susceptibility” genes, that may trigger the cascade of neurodegeneration interacting with environmental risk factors. Susceptibility genes could also play a role in fALS, (Zufiría et al., 2016). Mutations in susceptibility genes could potentially contribute to the development of the disease only in the presence of other genetic or environmental factors. In persons with a susceptibility genotype, the exposure to specific environmental risk factors may increase the risk for the disease.

Concerning genetic predisposition, epigenetics, and in particular defects in histone homeostasis (acetylation and deacetylation), have been implicated in ALS. Histone acetyltransferases (HATs) and histone deacetylases (HDACs) catalyze acetylation and deacetylation, respectively, of histone proteins Lys residues. The interplay between HATs and HDACs alters the net balance of histone acetylation levels, thereby remodeling chromatin structure, as indicated by transcriptional dysregulation that occurs in both ALS murine models and patients (Janssen et al., 2010). Although a role of epigenetic alterations in the pathogenesis of ALS has been documented, it remains to be clarified whether the involved epigenetic alterations could be related to environmental factors.

Among the risk factors for ALS, lifestyle, including smoking and dietary factors, and physical activity have been hypothesized. In particular, some ALS patients have a higher level of physical fitness and a lower body mass index compared with healthy controls (Armon, 2003; Turner, 2013). Moreover, environmental risk factors combined with working conditions (construction workers, carpenters, farm workers, laboratory technicians and athletes) could potentially increase the risk for ALS due to exposure to heavy metals, solvents, pesticides and chemicals which could contribute to trigger ALS pathogenetic mechanisms (Ingre et al., 2015).

In a geographical area which includes part of Japan, Guam, Kii Peninsula of Japan and Guinea the prevalence of the disease is 50–100 times higher than in any other part of the world. This increased incidence is ascribed to exposure to a neurotoxic amino acid, β-methylamino-L-alanine, as indicated by the higher concentration of this molecule in the brain and spinal cord tissues of ALS patients than in healthy controls (Pablo et al., 2009).

Viral infections have also been considered as a potential risk factor for ALS: exposure to viruses such as enterovirus, herpesvirus and retrovirus may play an important role in the disease, as reported in studies of blood, serum, muscle and post-mortem brain tissue of ALS patients. In these tissues a significant increase of the expression of the virus and the ability to form virus-like particles were revealed (Berger et al., 2000; Oluwole et al., 2007; Alfahad and Nath, 2013).

Another important factor to consider is the relationship between ALS and other medical conditions. Several studies suggest a strong association of head trauma, metabolic diseases, autoimmune pathology and neuroinflammation (Chen et al., 2007; Turner et al., 2013).

Altogether these data point to a substantial impact of non-genetic factors on ALS. The identification and knowledge of these factors would be highly relevant for understanding the etiopathogenesis of the sporadic form of the disease in persons with a susceptibility genotype.

About 5%–10% of ALS is familial, with a Mendelian pattern of inheritance. ALS can be inherited in an autosomal dominant, autosomal recessive or X-linked manner, although in most affected families it is inherited in an autosomal dominant manner. Patients with fALS present an earlier age of onset compared to the sporadic form, and the penetrance, severity, progression and duration of disease vary in different gene mutations and different mutations in the same gene (Chen et al., 2013).

More than 20 gene mutations have been identified in fALS, which include: Cu/Zn superoxide dismutase 1 gene (SOD1), TAR DNA-binding protein 43 (TDP43), fused in sarcoma (FUS)/translocated in sarcoma and ubiquitin 2 (Kaur et al., 2016). It has been recently reported that the hexarepeat expansion in chromosome 9 open reading frame 72 (C9orf72) is the most common inherited cause of fALS (40% of cases; Zufiría et al., 2016).

Mutations in the SOD1 gene were the first to be identified in ALS: they occur in up to 20% of fALS cases and in 1%–4% of sALS cases and, to date, more than 150 mutations have been found (Chen et al., 2013). Three different isoform of SOD metalloenzymes are encoded in the human genome: the cytoplasmic Cu/Zn SOD (SOD1), the mitochondrial Mn SOD (SOD2) and the extracellular Cu/Zn SOD (SOD3). Each isoform is a product of distinct genes and has a distinct subcellular localization, but all require metals for their activity and catalyze the same reaction: the dismutation of toxic superoxide anion radical (O_2_^−^), a reactive oxygen species (ROS) with a single unpaired electron produced by cellular respiration, into oxygen and hydrogen peroxide (H_2_O_2_). The toxicity of H_2_O_2_ is then removed by glutathione peroxidase or catalase, which generates water and oxygen. Therefore, SODs enzymes provide an important antioxidant defense in cells exposed to oxygen (Fukai and Ushio-Fukai, 2011).

The human SOD1 isoform, whose gene is located on chromosome 21 (locus 21q22.1), is a small gene consisting of five exons and four introns encoding for a homodimeric enzyme, each monomer formed by 153 amino acids (15.8 kDa) which form eight antiparallel beta strands. The protein contains one copper and one zinc atom: the first is fundamental for SOD1 activity, while zinc plays a role in the structural stability of the enzyme (Rosen et al., 1993).

The SOD1 mutations can be localized on the beta strand, determining structural protein instability, or in the metal-binding sites, determining the lack of Cu and/or Zn, conformational dysfunction and alteration of protein interactions. In both instances, the destabilization of the structure can lead to the formation of aggregates (Kaur et al., 2016).

The majority of SOD1 mutations are associated with an autosomal dominant form of the disease (ALS1) and G93A, alanine at codon 4 changed to valine (A4V), H46R and D90A are the most commonly reported ALS mutations. The G93A mutation (glycine 93 changed to alanine) is a rare mutation, but is the most studied as it was the first to be used in a transgenic mouse model of the disease. The A4V is the most prevalent mutation in the USA and D90A (aspartic acid at codon 90 changed to alanine) is the most common in Europe (with either dominant or recessive inheritance).

Gene mutations can cause a dominant gain of function, resulting in an increase of SOD1 activity, with an excessive production of H_2_O_2_, or in a dominant loss of function with a decrease in enzyme activity which results in insufficient degradation of ROS (Kaur et al., 2016).

The main pathogenetic mechanisms involved in motoneuron degeneration in ALS and the main therapeutic strategies proposed to date are here summarized. This review article also focuses on the application of mesenchymal stem cells (MSC) as treatment for ALS, reporting results obtained in *in vivo* models of the disease and in clinical trials. Moreover, extracellular vesicles (EVs) as possible mediators of a therapeutic effect of stem cells will be discussed, underlying their potential use for ALS treatment.

## Pathogenetic Mechanisms in ALS

**Figure 1:**
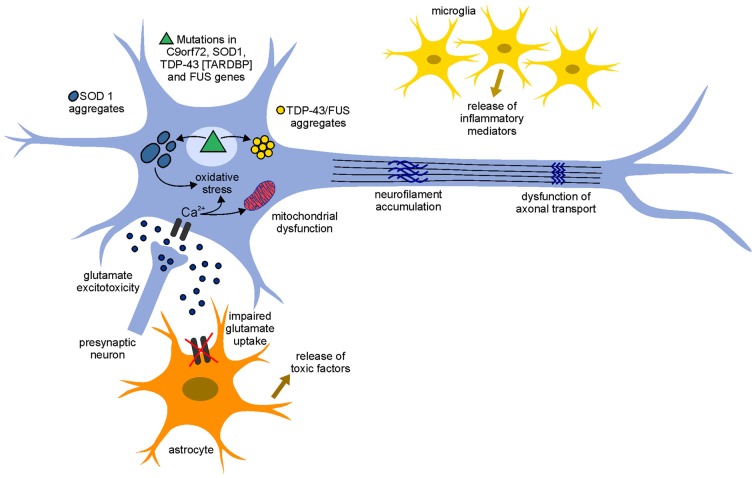
**Pathogenetic mechanisms involved in amyotrophic lateral sclerosis (ALS).** The pathophysiological mechanism of the disease appears to be multifactorial and several mechanisms contribute to neurodegeneration. An increase of the neurotransmitter glutamate in the synaptic cleft (glutamate excitotoxicity), due to the impairment of its uptake by astrocytes, leads to an increased influx of Ca^2+^ ions in the motoneurons. The increased levels of Ca^2+^ ions, which in physiological conditions could be removed by mitochondria (calcium homeostasis), remain high in the cytoplasm due to mitochondrial dysfunction and can cause neurodegeneration through activation of Ca^2+^-dependent enzymatic pathways contributing to oxidative stress. Mutant misfolding proteins (such as superoxide dismutase 1 gene (SOD1), chromosome 9 open reading frame 72 (C9orf72), TAR DNA-binding protein 43 (TDP-43) and fused in sarcoma (FUS) form intercellular aggregates, contribute to an increase of oxidative stress, contribute to mitochondrial dysfunction and could lead to the accumulation of neurofilaments (NFs) and dysfunction of axonal transport. Moreover, activated astrocyte and microglia release inflammatory mediators and toxic factors, contributing to neurotoxicity.

The identification of molecular mechanisms by which motoneurons degenerate in ALS is crucial for understanding disease progression and for the development of new therapeutic approaches. Although SOD1 mutations have been linked to ALS since more than two decades, the mechanisms underlying the mode of action of mutant SOD1 and the subsequent neurodegeneration/neurotoxicity are still unclear. Several hypotheses have been proposed in this regards and it seems likely that the combination of mechanisms, rather than a single mechanism, contributes to neurodegeneration in ALS, pointing to a multifactorial pathogenesis (Figure 1).

### Mitochondrial Dysfunction

Mitochondrial damage is a common feature of many neurodegenerative diseases. Mitochondria are the most important organelles for energy production, cellular respiration and calcium homeostasis. Moreover, they produce high level of ROS and play a key role in apoptosis, opening the permeability transition pore and allowing the release of cytochrome c, which leads to the activation of the caspase cascade. For these reasons, structural and biochemical alterations of mitochondria can be linked to many aspects of ALS pathogenesis.

Morphological alterations in mitochondria, such as vacuolated and dilated organelle with disorganized cristae and membranes, fragmented network and swelling, were observed in spinal motoneurons and skeletal muscle of both sALS and fALS patients and in the murine model of the disease (SOD1(G93A) mice; Boillée et al., 2006a; Sasaki and Iwata, 2007; Magrané and Manfredi, 2009). The formation of vacuoles is due to expansion of the mitochondrial intermembrane space and consequent distention of membranes (Higgins et al., 2003).

Although the mitochondria have own SOD protein (SOD2), the cytoplasmic SOD protein (SOD1) is also present, at low levels, in the mitochondrial intermembrane space and in their matrix (Bergemalm et al., 2006). The deposit of misfolded mutant SOD1 in mitochondria may alter the physiological function of these organelles in the cell metabolism. Abnormal production of ATP and ROS, dysfunction in energy homeostasis and calcium homeostasis, alteration of apoptosis triggering, as well as altered mitochondrial transport along axons have been reported in ALS transgenic mice and patients (Pasinelli et al., 2000; Mattiazzi et al., 2002; Menzies et al., 2002; Damiano et al., 2006). Concerning energy homeostasis and ATP deficits, mutant SOD1 causes a decreased activity of respiratory chain complexes I and IV which are associated with defective energy metabolism (Wiedemann et al., 1998). Another key function of mitochondria concerns the regulation of cytosolic calcium levels: several studies reported a loss of Ca^2+^ binding proteins in motoneurons of ALS patients related to the presence of mutant SOD1 (Bernard-Marissal et al., 2012; Mattson, 2013), which leads to reduced calcium uptake from the cytoplasm, increasing the sensitivity to excitotoxicity.

Moreover, mitochondria are required in areas with high demand of ATP and calcium homeostasis, such as synaptic terminals. Therefore, transport of mitochondria to these areas is of primary importance, and defects in mitochondrial axonal transport lead to metabolic alterations in neurons (Magrané and Manfredi, 2009; Mòrotz et al., 2012).

Altered protein expression in mutant mitochondria has also been reported (Fukada et al., 2004; Kirby et al., 2005; Lukas et al., 2006). Since the majority of proteins required for the function of this organelle are synthesized in the cytoplasm and imported in the mitochondria, mutant SOD1 associated to the mitochondrial surface could prevent protein import (Liu et al., 2004).

### Glutamate Excitotoxicity

Glutamate, the main excitatory neurotransmitter in the central nervous system (CNS), is synthesized in the presynaptic terminal and diffuses across the synaptic cleft, activating specific postsynaptic receptors and triggering action potentials. This neurotransmitter acts on different receptors on the dendrites of the postsynaptic motoneuron, such as α-amino-3-hydroxyl-5-methyl-4-isoxazole-propionate (AMPA) and N-methyl-D-aspartate (NMDA) receptors. The depolarization of neuronal membranes after activation of neuronal glutamate receptors activates voltage-dependent calcium channels, allowing calcium to enter the cell.

After release from the presynaptic neuron, glutamate is removed from the synaptic cleft by several glial and neuronal cell transporter proteins, excitatory amino acid transporters (EAATs; Sundaram et al., 2012). The concentration of glutamate in the synaptic cleft is thus finely regulated, avoiding excitotoxicity: an excessive or prolonged activation of glutamate receptors results in degeneration and eventually death of the involved neurons (Shaw and Eggett, 2000). A sustained elevation of intracellular calcium levels triggers enzymatic and mitochondrial damages that lead to the formation of ROS and activates several destructive biochemical processes, determining excitatory damage and neuronal degeneration (Ilieva et al., 2009; Vucic et al., 2014). Therefore, the rapid removal of glutamate is required to prevent neuronal toxicity. In particular, the isoform 2 of the astroglial glutamate transporter (EAAT2) is involved in keeping the amount of glutamate below excitotoxic level in the nervous system.

Before the identification of the genetic links of fALS, glutamate excitotoxicity was one of the first hypotheses proposed as pathogenetic mechanism of ALS (Bendotti and Carrì, 2004). The motor cortex and spinal cord of ALS patients and transgenic mutant SOD1 mouse models were found to have reduced EAAT2 level, probably due to the presence of aberrant EAAT2 mRNA or to cleavage of the EAAT2 transporter. This leads to an increase of synaptic glutamate concentration and an over-stimulation of glutamate postsynaptic receptors, determining excitotoxic neuronal degeneration (Lin et al., 1998; Zarei et al., 2015). The loss of functional EAAT2 was also observed in affected brain regions of other neurodegenerative diseases, such as Alzheimer’s and Huntington’s diseases (Guo et al., 2003). To establish whether loss of functional EAAT2 in ALS was a primary cause of neuron degeneration or a consequence of cell damage, transgenic mutant SOD1 mice overexpressing EAAT2 (EAAT2/G93A double transgenic mice) were generated to investigate whether supplementation of EAAT2 loss would delay or rescue the disease. Mice with an increased EAAT2 expression showed a delay in motoneuron degeneration and disease progression. Since no change in lifespan was found comparing EAAT2/G93A mice with SOD1(G93A) mice, it was concluded that loss of EAAT2 may contribute to motoneuron degeneration in ALS but is not the principal cause (Guo et al., 2003).

Moreover, the selective vulnerability of motoneurons in ALS may be due to a higher permeability to calcium of these cells compared with other neurons, probably caused by a defect in RNA editing of the GluR2 subunit of AMPA, which renders this receptor more permeable to calcium (Kawahara et al., 2004).

### Oxidative Stress

Free radicals or ROS are natural products of oxygen metabolism. The term oxidative stress is used when the production of ROS is higher than the capacity of cells to remove them. This leads to the accumulation of ROS, which causes irreversible damage to cell structures and macromolecules, such as proteins, DNA and RNA.

SOD1 is the major enzyme to prevent oxidative damage and to reduce superoxide leakage from mitochondria. Mutations in this gene can cause alterations in the activity of the protein leading to cytotoxicity.

Early studies suggested that mutations in the SOD1 gene cause a complete loss of protein function (Deng et al., 1993). Subsequently, *in vitro* experiments demonstrated that mutant human SOD1 proteins (such as the G37R mutation) are active and stable, promoting neural apoptosis in a dominant manner (Borchelt et al., 1994; Rabizadeh et al., 1995). These studies suggest ALS pathogenesis may involve not only a decrease/loss in the enzymatic function of SOD1, but is also probably due to a dominant toxic gain of function of the enzyme. It has been proposed that mutant SOD1 could revert its normal antioxidant activity producing toxic superoxide: the mutated protein could take electrons from other cellular antioxidants and donate them to molecular oxygen, producing superoxide and making SOD1 the source of oxidative stress (Beckman et al., 2001; Liochev and Fridovich, 2003). Increased levels of free radicals and of oxidative damage were found in cerebrospinal fluid (CSF), serum and urine samples of ALS patients (Zarei et al., 2015) and could be due to an altered geometry in the active site of the mutated SOD1, which allows entry of reducing substrates.

Oxidative stress in ALS may also derive from a defective oxidative phosphorylation (Bacman et al., 2006), as reported from studies of CSF in transgenic mice and in patients, in which ROS produced from defective oxidative phosphorylation, such as 3-nitrotyrosine, were found in high concentration (Tohgi et al., 1999). Moreover, this study combined the increase of ROS with mitochondrial dysfunction and provided an example of how different pathogenetic mechanisms of ALS may be inter-related.

### Protein Aggregates

Protein aggregates are a pathological hallmark of many neurodegenerative diseases such as Alzheimer’s, Parkinson’s, Huntington’s diseases and ALS. These aggregates derive from the accumulation of misfolded proteins, which oligomerize and aggregate, gaining toxic properties (Julien, 2001). Inclusions rich in mutated SOD1 proteins have been found in tissues from both sALS and fALS cases, as well in mutant SOD1 transgenic mice (Boillée et al., 2006a).

The structure of SOD1 in the aggregates is not clear, but it seems that the protein is disulfide-reduced and lacks both copper and zinc atoms. The aberrant accumulation of mutated proteins is also related to a lack of their degradation, resulting in aggregates consisting of mutated SOD1 and other mutated proteins related to ALS, such as TDP43 or FUS.

TDP43, an RNA-binding protein, is normally located in the nucleus, where it regulates transcription, splicing and mRNA transport. This protein is necessary for the prevention of DNA damage (Hill et al., 2016). TDP43 aberrant protein inclusions have been reported in 80% of ALS cases and it seems that the cytoplasmic accumulation is due to mutations in the 3′ UTR region of the genes, which lead to overexpression and altered location of the protein (Neumann et al., 2006; Coan and Mitchell, 2015). As TDP43, FUS is a nuclear protein. The accumulation of TDP43 and FUS in the cytoplasm is probably due to mutations which prevent their shuttles to the nucleus (Dormann and Haass, 2011).

Protein inclusions in ALS contain also other components, such as chaperones, mitochondrial proteins, ubiquitin and neurofilaments (NFs). Moreover, the aberrant accumulation of ubiquitin and of ubiquinated and misfolded proteins could affect the normal function of the proteasome machinery, impairing normal protein degradation and leading to further protein accumulation, degeneration and death of motoneurons.

### Accumulation of Neurofilaments

Accumulation and/or aggregation of NFs in the cell bodies and axons, and abnormal location of phosphorylated NFs in the cell body are typical pathological hallmarks of ALS. NFs are the major intermediate filaments in neurons; they are the most abundant cytoskeletal components of large myelinated axons and control the axonal caliber. NFs are formed by the co-polymerization of light (NF-L, 65 kDa), medium (NF-M, 95 kDa) and heavy (NF-H, 115 kDa) subunits. NF-L is necessary for filament assembly, whereas NF-M and NF-H form links with other NFs in the axon (Julien, 1999). Also α-internexin is an integral component of NFs in the CNS, in particular during axon elongation (Yuan et al., 2006).

The mechanisms leading to the formation of NF aggregates in ALS are still unclear. Mutations in NF genes occur in fALS and sALS and seem to be correlated with abnormal phosphorylation of NFs. Phosphorylation of NF-H and NF-M usually occurs only in the axon and the rate of NF transport is inversely correlated to their phosphorylation state. The abnormal phosphorylation could alter the axonal transport of NFs, determining their accumulation in the cell bodies and proximal axon. This accumulation could be at the basis of defects in axonal transport of other cellular components important for cell survival, such as mitochondria (Xiao et al., 2006).

The aggregation of NFs could also be due to their altered stoichiometry: overexpression or downregulation of NF subunits in murine models of ALS provoke NF accumulation (Perrot and Eyer, 2009). Transgenic SOD1(G93A) mice which overexpress the NF-L subunit exhibit excessive accumulation of NFs in the perikarya and proximal axon of motoneurons in the ventral horn of the spinal cord, accompanied by proximal axonal swelling and subsequent degeneration (Xu et al., 1993). *In situ* hybridization revealed a consistent reduction in NF-L mRNA levels in degenerating spinal motoneurons of ALS patients (Tomkins et al., 1998; Al-Chalabi et al., 1999; Wong et al., 2000), while NF-L deficiency accelerates motoneuron degeneration in transgenic mice (Xu et al., 1993), indicating the importance of NF protein stoichiometry in the distribution and aggregation of NFs.

Surprisingly, overexpression of both NF-L and NF-H subunits significantly slowed down disease progression in mouse models of ALS. Double transgenic mice (SOD1(G93A) gene and NF-L or NF-H genes) showed a significant delay in disease progression and an extension of survival compared with SOD1(G93A) mice (Kong and Xu, 2000), suggesting a protective effect of NF accumulation. The mechanism of this protection is unclear, but perikaryal accumulation of NFs rather than their axonal deficiency could be responsible in slowing down the disease.

The abnormal organization of NF seems to be involved in the pathogenesis of ALS, although the relationship between their accumulation and motoneuron neurodegeneration remains unclear. Despite the correlation of some mutations of NF genes with ALS disease, specific mutations in NF genes have not been identified, indicating that probably NF gene mutations are not a common cause of ALS but could represent a risk factor for selective motoneuron vulnerability.

### Neuroinflammation

A common characteristic of ALS and other neurodegenerative diseases is the neuroinflammatory response, characterized by activated microglia, astrogliosis and infiltrating immune cells in the sites of neuronal injury. Although ALS involves the selective death of motoneurons, different lines of evidence have shown that neuronal injury is non-cell-autonomous but depends on a finely regulated dialog between motoneurons and glial cells. Deregulated communication between neurons and glial cells compromises neuronal homeostasis and survival, and the involvement of mutant glia in motoneuron degeneration is well documented. The expression of mutant SOD1 gene limited to motoneurons is not sufficient to cause disease in transgenic mouse model of ALS, leading to suppose that their degeneration requires the participation of non-neuronal cells (Clement et al., 2003). Non-mutated motoneurons surrounded by glial cells carrying a SOD1 mutated gene develop the pathological phenotype, while mutated motoneurons surrounded by wild-type glia show a healthy phenotype (Clement et al., 2003). Moreover, the replacement of mutant SOD1 in glial cells with wild-type glia delays the disease and prolongs the survival of ALS mice (Lee et al., 2012). Thus, damaged glia and neurons act together contributing to the process of neurodegeneration and to disease progression.

In the CNS, microglial cells are the resident macrophages and represent the first line of defense against infection or injury, monitoring the extracellular environment and interacting with neurons and astrocytes. These cells have immunological properties, with both neuroprotective and neurotoxic potential. After CNS injury, microglial activation is a major component of neuroinflammation. In particular, in ALS the interaction between motoneurons and microglia initially protects neurons. When motoneuron damage worsens, motoneurons and astrocytes release misfolded proteins (such as mutated SOD1) and other toxic molecules that stimulate the activation of microglial cells, which switch from an anti-inflammatory and neuroprotective to a pro-inflammatory and neurotoxic phenotype (Appel et al., 2011; Zhao et al., 2013). Microglial activation includes activated microglia (M1) and alternatively activated microglia (M2). M1 microglia are cytotoxic and secrete ROS, proinflammatory cytokines and neurotoxic molecules, mediating motoneuron death (Almer et al., 1999; Elliott, 2001). Studies in SOD1 transgenic mice have shown that the replacement of mutant SOD1 microglia with wild-type microglia, as well as the specific reduction of the expression of mutant SOD1 gene in these cells, significantly reduce motoneuron degeneration and extend the lifespan of the animals (Beers et al., 2006; Boillée et al., 2006b). This points to an involvement of microglial cells in neurodegeneration, underlining that mutant SOD1 microglia acquire a M1 toxic phenotype promoting disease progression.

Unlike the M1 phenotype, M2 microglia produce high levels of anti-inflammatory cytokines and neurotrophic factors that enhance the protection and survival of motoneurons. The upregulation of M2 markers in the spinal cord of SOD1(G93A) mice demonstrated that in the early stage of the disease microglia display an M2 phenotype which promotes repair and regeneration. During disease progression, damaged motoneurons induce microglial cells to release ROS and proinflammatory cytokines and to acquire an M1 phenotype leading to further neurotoxicity (Zhao et al., 2013). Activated microglia increase during disease progression due to their interaction with the cell microenvironment at different stages of the disease, which plays a key role in determining a neuroprotective or neurotoxic function of microglia.

Astrocytes are the largest glial cell components of the CNS and play many important functions in maintaining and supporting neurons. Reactive astrogliosis has been implicated in neurodegeneration and in the progression of ALS. One of the most important functions of astrocytes in supporting neurons is to maintain low concentration of glutamate in the synaptic cleft through EAAT2 glutamate receptors. In patients with sALS and fALS, as well as in SOD1 mice, astrocytes downregulate the EAAT2 transporter, determining a less efficient uptake of glutamate and contributing to excitotoxicity (Howland et al., 2002). Other studies have shown that, upon activation, an insufficient release of neurotrophic factors and the release of neurotoxic factors from astrocytes are involved in neurodegeneration (Komine and Yamanaka, 2015). This was also indicated by analyses of astrocytes in post-mortem tissue of ALS patients, which revealed an upregulation of 22 genes encoding chemokines, proinflammatory cytokines and components of the complement cascade, which could exacerbate neural damage and loss of already compromised neurons (Zhao et al., 2013). Moreover, the selective removal of the mutant SOD1 gene from astrocytes, or the transplantation of healthy astroglial cells, slowed down the disease progression, attenuated motoneuron loss and increased the lifespan in SOD1 transgenic mice (Lepore et al., 2008; Yamanaka et al., 2008), indicating the involvement of astroglial cells in the neurodegeneration process.

## Therapeutic Approaches in ALS

Given the complexity of ALS pathogenesis, to date there is no effective treatment to cure or significantly ameliorate the quality of life of patients. Nevertheless, several therapeutic strategies have been proposed to relieve symptoms and improve the quality of life of ALS patients.

### Pharmacological Therapy

The only drug approved by the US Food and Drug Administration is riluzole (2-amino-6-trifluoromethoxy benzothiazole, also known as rilutek), that acts as an inhibitor of glutamate release from the presynaptic terminals by blocking voltage-gated sodium channels, thus limiting glutamate excitotoxicity. Moreover, riluzole has other neuroprotective pharmacological actions, including the modulation of the NMDA ionotropic receptors, inactivation of voltage-dependent sodium channels and inhibition of the uptake of the inhibitory neurotransmitter γ–aminobutyric acid (Gurney et al., 1998). *In vitro* studies have demonstrated that riluzole protects motoneuron cell lines from glutamate stress, blocking excitotoxic damage (Doble, 1997). Neuroprotection has also been observed *in vivo* in models of ALS, in which treatment with the drug before the onset prolongs survival and slows neurodegeneration, improving motor performance of the treated animals (Gurney et al., 1998). However, the clinical use of riluzole (100 mg daily) prolongs the life of patients by 3 months, although the results obtained in clinical trials are often controversial (Miller et al., 2012). The beneficial effect is very modest: only a small beneficial effect in both bulbar and limb functions but no effect on muscle strength were found (Musarò, 2013). In some studies, the neuroprotective effect of riluzole was examined in combination with other drugs. Rasagiline is an antiapoptotic drug which reduces oxidative stress (by inhibition of monoamine oxidase B) and preserves mitochondrial membrane potential. The combined treatment of rasagiline with riluzole determined a dose-dependent improvement in motor performance and extended survival in SOD1(G93A) mice more than treatments in which rasaligine was administered alone (Waibel et al., 2004). Given the modest efficacy and the high cost, doubts persist about the clinical administration of these drugs and additional studies are needed to improve their clinical effect.

Since loss of neurotrophic support to motoneurons has been proposed as mechanism contributing to ALS, several studies have reported that increased levels of growth factors (such as insulin-like growth factor 1, glial cell line derived growth factor and ciliary neurotrophic factors) have a positive effect in experimental models of the disease (Gould and Oppenheim, 2011), promoting neuron hypertrophy and survival. In particular, in animal models, the efficacy of growth factors delivered by viral-mediated gene (to stimulate the secretion of specific trophic factors to target cells) or by directed infusion of the trophic factors in the spinal cord or in the brain provided a modest benefit slowing down disease progression and increasing survival of the animals (Boillée et al., 2006a). However, human trials based on the use of neurotrophic factors showed modest or no effects (Beghi et al., 2011).

These pharmacological strategies are directed against one or a few altered mechanisms involved in ALS and their use has only a minimal impact on the disease course. Probably, for an efficient therapeutic approach it could be helpful to counteract different pathogenetic mechanisms involved in the disease. For this reason, since the transplantation of stem cells and gene therapy can act via multiple mechanisms, in the last years an increasing interest has been addressed to these therapeutic approaches.

### Gene Therapy

ALS can be caused by dominant mutations in SOD1 that give toxic property to the mutant protein. Since the complete absence of the SOD1 gene does not cause the disease in mice (Reaume et al., 1996), the SOD1 gene silencing has been regarded as an interesting possibility to limit the course of the pathology. In particular, a lentivirus encoding for a RNA silencing (siRNA) that catalyzes the selective degradation of SOD1-mRNA was used in this approach. The injection of this virus in the muscle or directly in the spinal cord of transgenic mouse models of ALS reduced SOD1 expression delaying the neurodegeneration, but data about the slowing down of the disease progression and survival are controversial (Ralph et al., 2005; Raoul et al., 2005). Moreover, this approach could be used only for a familial form of ALS and not for sALS which represents the majority of cases.

An antisense oligonucleotide directed to SOD1-mRNA was found to decrease the concentration of SOD1 mRNA and related protein in the spinal cord, prolonging survival in the SOD1(G93A) rat model of the disease (Smith et al., 2006). After this encouraging result, to date the only clinical trial performed in phase I has shown the safety, tolerability and pharmacokinetics of this antisense oligonucleotide (Miller et al., 2013). Despite the small number of patients, the study showed that the intrathecal administration of antisense oligonucleotide is safe and well-tolerated. However, antisense oligonucleotides do not cross the blood-brain barrier (BBB) and, to cure ALS patients, they should be delivered directly to the CNS (O’Connor and Boulis, 2015), which is difficult to achieve. Moreover, this type of treatment requires a constant infusion of repeated doses of the antisense nucleotide. Gene therapy requires to be optimized for a successful approach to neurodegenerative diseases.

### Stem Cell Therapy

Stem cells are becoming a promising therapeutic approach to neuronal replacement and regeneration, becoming a source of great hope and expectation for patients affected by different neurodegenerative diseases, including ALS. The use of stem cells as therapeutic approach is of great interest given their ability to home to damaged sites, stimulate tissue repair and regeneration, and direct their differentiation in response to extracellular signals (Faravelli et al., 2014).

Stem cells are a group of cells with the ability of self-renewal and to differentiate in many cell types. They are usually divided in two major subtypes: embryonic stem cells (ESC), originated from the inner cell mass of blastocyst and that possess the ability to produce all the three germ layers; adult stem cells, specialized cells that can differentiate into many cell types of different organs, usually determined by the germ layer of origin (Meamar et al., 2013). Among the different stem cell types, the most common used for neurological diseases are: ESC, neural stem cells (NSC), MSC and induced pluripotent stem cells (iPSC). The best source of stem cells should be chosen on the basis of their biological features to survive, migrate to the damaged tissues, engraft and differentiate (Faravelli et al., 2014).

The main objectives of the treatment with stem cells are cellular replacement and neural protection. The first objective would be achieved by the stem cell differentiation into specific cell subtypes involved in the disease, with the purpose to replace dead cells. The second objective involves the use of stem cells based on their ability to release trophic factors and to remove neurotoxic molecules, providing a local support in the microenvironment of the damaged area, acting as neural protectors.

Among the different types of stem cells used in the treatment of neurodegenerative diseases, MSC seem to have aroused the highest interest as promising candidates (Baglio et al., 2012). MSC can be isolated from a variety of fetal and adult tissues, such as skeletal muscle, placenta, umbilical cord, blood and adipose tissue. Adipose tissue is gaining increasing interest because it is available in large amount from liposuction, allowing autologous transplantation of adipose-derived MSC (ASC).

There are several technical advantages in the application of MSC. First of all, their isolation is safe and easy, their expansion *in vitro* is simple and it was demonstrated, both *in vitro* and *in vivo*, that they can differentiate in neural-like, glial-like and astrocytic-like cells (Marconi et al., 2013; Haidet-Phillips and Maragakis, 2015). MSC are less susceptible to tumoral changes and do not require immunosuppressive treatment to prevent rejection given the possibility of autologous transplantation. Moreover, as the other stem cells, after transplantation and migration, MSC are attracted to the damaged area, where they increase the release of neurotrophic factors (Meamar et al., 2013).

Several studies have provided evidence for the efficacy of MSC in *in vivo* models of ALS, demonstrating that their injections can delay the death of motoneurons, decrease the inflammatory response and may prolong survival of the animals. Results of some preclinical studies in *in vivo* models of ALS with MSC are reported in Table 1.

**Table 1:** **Applications of mesenchymal stem cells (MSC) in amyotrophic lateral sclerosis (ALS) models**.

ALS model	Stem cell type	Injection specifics	Results	References
SOD1(G93A) mouse	hBM-MSC	Intraspinal (10^5^ cells) Presymptomatic (week 28)	Improvement of motor performance, migration and engraftment of MSC in the spinal cord, prevention of astroglial and microglial activation	Vercelli et al. (2008)
SOD1(G93A) rat	rHSC	Intrathecal (2 × 10^6^ cells) Clinical onset (week 12)	Reduction of motoneurons death and inflammation, improvement of motor functions and extended survival	Boucherie et al. (2009)
SOD1(G93A) mouse	hBM-MSC	Intrathecal (10^6^ cells) Presymptomatic (week 8)	Improvement of motor performance, reduction of motoneuron death and prolonged lifespan	Kim et al. (2010)
SOD1(G93A) rat	rMSC	Intraspinal (10^5^ cells) and intravenous (2 × 10^6^ cells) Clinical onset (week 16)	Improvement of motor performance, increase of survival, migration and engraftment of MSC in the spinal cord	Forostyak et al. (2011)
SOD1(G93A) mouse	hBM-MSC transfected with GLP-1	Intracerebroventricular (2.8 × 10^3^ cells) Presymptomatic (week 5)	Improvement of motor performance, delay of disease onset and survival	Knippenberg et al. (2012)
SOD1(G93A) mouse	mBM-MSC	Intravenous (3 × 10^6^ cells) Clinical onset (week 12)	Improvement of motor functions and survival. Reduction of oxidative stress; limited migration and engraftment of MSC in the spinal cord	Uccelli et al. (2012)
SOD1(G93A) mouse	mASC	Intravenous (2 × 10^6^ cells) Clinical onset (week 11)	Improvement of motor functions, delay of motoneuron death, limited migration and engraftment of MSC in the spinal cord, modulation of neurotrophic molecules.	Marconi et al. (2013)
SOD1(G93A) mouse	hBM-MSC	Intracisternal (3 × 10^5^ cells) Clinical onset (week 16–18)	Delay of motoneuron death, reduction of astrogliosis, modulation of microglial activation, increase of IL-13 expression	Boido et al. (2014)

Due to the positive results obtained in *in vivo* models of the disease, in the last years numerous cell-based clinical trials for ALS have used MSC, and have reported the feasibility, safety and immunological effects of administration of MSC to ALS patients (Table 2).

**Table 2:** **Clinical applications of MSCs in ALS patients**.

Stem cell type and specifics	Delivery method/Cell number	Trial status and details	References
Autologous BM-MSC	Intraspinal (14–60 × 10^6^ cells)	The approach is safe and feasible. Some patients demonstrate electroneuromyography improvements	Deda et al. (2009)
Autologous BM-MSC	Intrathecal (54.7 × 10^6^ ± 17.4 × 10^6^ cells) Intravenous (23.4 ± 6 × 10^6^ cells)	The approach is safe, feasible and induces immediate immunomodulatory effects. Phase I complete, Phase II open to evaluate preliminary effects at various doses of cells	Karussis et al. (2010)
Autologous BM-MSC	Intraspinal (15–110 × 10^6^ cells)	The approach is safe and feasible, with no signs of toxicity, adverse events or abnormal cell growth. Phase I complete; no long-term harmful consequences, however disease progression did not appear to be slowed	Mazzini et al. (2010); Mazzini et al. (2012)
Autologous BM-MSC	Intraspinal (138–602.87 × 10^6^ cells)	The approach is safe and feasible. No acceleration in decline noted and an increase in spinal cord motoneurons numbers were identify after autopsy	Blanquer et al. (2012)
Autologous BM-MSC	Intraventricular (1 × 10^6^ cells/kg)	The approach is safe and feasible	Baek et al. (2012)
Autologous BM-MSC	Motor cortex (2.5–7.5 × 10^5^ cells)	The approach is safe and feasible, with a higher significant survival in treated patients	Martinez et al. (2009); Martínez et al. (2012)
Autologous BM-MSC and neural-induced MSC	Intravenous (42–102 × 10^6^ cells) and intralumbar (5–9.7 × 102 × 10^6^ cells)	The approach is safe and delay the disease progression, improving the quality of life of patients	Rushkevich et al. (2015)
Autologous BM-MSC	Intrathecal (1–2 × 10^6^ cells) Intramuscular (1–48 × 10^6^ cells)	The approach is safe and feasible. Phase I complete, Phase II open to evaluate preliminary effects at various doses of cells	Petrou et al. (2016)

However, despite stem cell-mediated therapy represents a new promising approach to cure ALS and other neurodegenerative disorders, to date no significant effects on the disease progression have been obtained compared to placebo-treated controls and key issues need to be solved before extensive clinical translation. First, it is necessary to identify the delivery of the cells to the CNS using standardized protocols and considering the route of administration, the dose of cells, the timing and the number of cell-injections. Moreover, given the high proliferative capacity of stem cells, the oncogenic transformation of transplanted cells should be prevented and their immunorejection avoided.

After the demonstration that only a small proportion of injected stem cells can reach, engraft and differentiate in the site of injury, the idea that the beneficial effect of stem cells is indirect and most probably depends on their paracrine activity is becoming more pervasive (Baglio et al., 2012; Maumus et al., 2013; Lai et al., 2015). Stem cells produce a large spectrum of EVs which contain cytokines, chemokines, growth factors, proteins and nucleic acids, and can exert significant effects on cells, mediating cell-cell communication. These observations led to the hypothesis that stem cells exert their beneficial effect through the secretion of EVs which enhance the repair of the damaged area releasing their content. Thus, EVs could be used as a novel cell-free therapeutic approach, avoiding all the risks associated with the use of cells.

## Extracellular Vesicles

Intercellular communication plays a fundamental role in multicellular organisms. It can be mediated through a direct contact between cells or by the secretion of molecules, such as growth factors, chemokines and cytokines, exerting their effect on cells in their proximity. In the last decades, a novel mechanism of cell communication has been proposed and involves intercellular transfer of EVs. These vesicles represent a vehicle to transfer membrane and cytoplasmic proteins, lipids and nucleic acids between cells, becoming an integral part of the intercellular microenvironment and playing a role in many physiological and pathological processes.

EVs are spherical particles enclosed by a phospholipid bilayer. They can be distinguished on the basis of their size (ranging from 30 nm to 1000 nm in diameter), lipid and protein composition, sedimentation rate, flotation density on a sucrose cargo and biogenesis pathway (Maumus et al., 2013; Lai et al., 2015). According to the biogenesis, morphology, protein compositions and size, EVs are classified as microvesicles and exosomes.

### Microvesicles

Microvesicles (also known as shedding vesicles) range from 150 nm to 1000 nm in diameter, although the size ranges of microvesicles and exosomes may overlap, especially when vesicles are isolated from biological fluids (György et al., 2011). Microvesicles are released by budding from the plasma membrane through a process dependent on intracellular calcium concentration, calpain and cytoskeleton reorganization, unlike exosomes, whose release is independent of cell calcium influx (Maumus et al., 2013). Calcium ions are responsible for the changes in phospholipid distribution in the plasma membrane, which is of primary importance for the formation of cytoplasmic protrusions (Biancone et al., 2012). Microvesicles are surrounded by a phospholipid bilayer and contain proteins, lipids and nucleic acids depending on the cell of origin. A typical characteristic of these vesicles is that they usually contain proteins associated with lipid rafts and cholesterol, sphingomyelin, ceramide and phosphatidylserine (Biancone et al., 2012).

### Exosomes

Exosomes are EVs produced by almost all cell types, with a diameter ranging from 30 nm to 100 nm and a flotation density of 1.10–1.18 g/ml (Lai et al., 2015). They are present in many biological fluids, including urine, saliva, blood, amniotic fluid and CSF, as well as in a conditioned medium of cell culture (Kourembanas, 2015). The most commonly used techniques for their isolation are represented by ultracentrifugation, ultrafiltration and immunoprecipitation, although many isolation kits are currently available (Maumus et al., 2013; Raposo and Stoorvogel, 2013).

The morphology of exosomes is described as “cup shaped” after visualization by transmission electron microscopy. They are surrounded by a phospholipid bilayer and contain many characteristic proteins, lipids and functional RNAs, in particular mRNAs and microRNA (miRNAs) that can be transferred between cells. Concerning protein composition, exosomes from different cell types present typical endosome-associated proteins (as GTPase and Alix) or membrane proteins (as tetraspanin CD63, CD9, CD81 and heat-shock proteins Hsp70 and Hsp90) involved in their biogenesis, transport and fusion (Raposo and Stoorvogel, 2013).

The cargo and composition of exosomes are unique and depend in part from the cell of origin and its physiological state (Jarmalavičiūtė and Pivoriūnas, 2016). Exosomes derived from diseased cells are usually implicated in the spread of pathological processes or in the propagation of diseases, while exosomes derived from stem cells can be used as therapeutic tools (Jarmalavičiūtė and Pivoriūnas, 2016). Many studies on the biochemical composition of exosomes have been performed. The results on the composition of exosomes in terms of proteins, nucleic acids and lipids are reported in the database ExoCarta^1^, recently incorporated in another database, Vesiclepedia, which includes data from all types of EVs (Mathivanan et al., 2012; Raposo and Stoorvogel, 2013).

Some of the properties (such as size and morphology) of exosomes overlap with those of microvesicles, but exosomes are the only class known to have an endosomal origin, distinguishing them from other vesicles. Exosomes originate from invagination and endocytosis of the plasma membrane. These early endosomes mature through a series of transformation and changes in protein content to become late endosomes. The membranes of late endosomes give rise to vesicles ranging from 30 to100 nm in size (exosomes), contained in multivesicular bodies which during this phase have incorporated proteins (from plasma membrane and Golgi complex), lipids and nucleic acids (Zhang et al., 2016). The multivesicular bodies can be degraded in lysosomes or can be fused with the plasma membrane, releasing exosomes in the extracellular environment.

Released exosomes can act in a paracrine or endocrine manner, modifying the behavior of adjacent or distant cells. The role of exosomes in physiological and pathological processes depends on their ability to interact with recipient cells to deliver their content. In particular, the vesicles can be internalized by recipient cells by endocytosis, by fusion or after contact with surface receptors (Raposo and Stoorvogel, 2013; Kourembanas, 2015).

### Functions of Extracellular Vesicles in Intercellular Communication

Once secreted from the cells, exosomes and microvesicles can interact with adjacent or distant recipient cells and deliver their cargo, modulating their activities. In addition to providing biologically active molecules, EVs protect these molecules against degradation until they reach the target site, and facilitate their uptake from the recipient cells. The uptake can be direct, by fusion with the target cell or by endocytosis, or indirect, mediated by receptor binding. The fusion of EVs with the plasma membrane determines the direct release of the content of EVs inside the cells, while, with the other mechanisms, EVs remain within the endosome until they fuse with the endosome membrane, releasing their content into the cytoplasm. Alternatively, endosomes can fuse with lysosomes, leading to the degradation of the vesicles and their content (Turturici et al., 2014).

The proteins and functional RNAs contained in the EVs are implicated in many biochemical and cellular processes, such as communication, inflammation, tissue repair and regeneration, cell differentiation, as well as metabolism (Lai et al., 2015).

The cargo, and therefore the functions, of EVs depend on the phenotype of the cell of origin. Nevertheless, it has been shown that a selective enrichment of specific molecules occurs: some proteins and, above all, functional RNAs are preferentially sorted to EVs and released through them (Li et al., 2013) and the selective fate of molecules into the EVs is finely regulated in both physiological and pathological conditions. During development, secreted EVs contain morphogens (such as sonic hedgehog and retinoic acid), playing an important role in developmental signaling and morphogenesis (Greco et al., 2001). EVs also contain bioactive lipids that can stimulate cytokine secretion, induce chemotaxis or inhibit apoptosis (Turturici et al., 2014). EVs released from tumor cells contain molecules which stimulate proliferation, angiogenesis and metastasis (Turturici et al., 2014). Moreover, in this case, cells release EVs containing caspase-3 and Fas ligand, allowing tumor cells to escape from apoptosis and immunosurveillance.

Concerning EVs secreted from stem cells, it was hypothesized that they can be involved in self-renewal and the pluripotency typical of these cells, and that they can play key roles in signaling within the stem cell niche.

Recently, more attention has been given to the ability of EVs to transfer genetic information between cells, in particular mRNAs and miRNAs. Intercellular communication based on these functional RNAs require the selectively compartmentation of RNAs in an appropriate vehicle, the protection from circulating RNAses, and the ability of RNA to retain the capability to control gene expression. For these reasons, EVs represent an optimal candidate for genetic exchange between cells, modulating the behavior of recipient cells. Exosomes released from MSC may regulate neurite outgrowth and neural plasticity by the transfer of miRNA-133b (Xin et al., 2012). Exosomes obtained from bone marrow-MSC (BM-MSC) contain miRNA-21 and miRNA-222 which are involved in cell proliferation and differentiation (Collino et al., 2010). This indicates that the biological effects on the neighboring cells can be, in part, regulated by shuttled-RNA of EVs.

### Therapeutic Applications of Exosomes

Since exosomes and microvesicles can transfer biological information over long distance, increasing attention has been paid to these vesicles as promoters of suppressors of pathological processes. As exosomes are smaller than microvesicles and capable to cross the BBB, here we will focus on the use of exosomes as a potential therapeutic tool in neurodegenerative diseases, such as ALS, Alzheimer’s and Parkinson’s diseases.

There is evidence that infusion of exosomes isolated from neuroblastoma or primary neurons into the brain ameliorates Alzheimer’s disease course in a mouse model, sequestering intracerebral amyloid-β (Aβ) peptide (Yuyama et al., 2015).

Another cellular source to obtain exosomes is represented by MSC and several studies have demonstrated the neuroprotective effects of these exosomes. In particular, exosomes derived from MSC isolated from dental pulp protected dopaminergic neurons against neurodegeneration induced by 6-hydroxy-dopamine oxidative stress, reducing the production of ROS and consequently apoptosis, indicating a potential therapeutic use of exosomes in the treatment of Parkinson’s disease (Jarmalavičiūtė et al., 2015). Another study reported that exosomes isolated from adipose tissue-MSC decrease the levels of Aβ in an *in vitro* model of Alzheimer’s disease, thanks to the high level of neprilysin (an important protein for the proteolysis of Aβ, whose activity and expression decrease during the disease) contained in the exosomes (Katsuda et al., 2013). The neuroprotective effect of exosomes obtained from ASC was also demonstrated in human neuroblastoma cell line and primary murine hippocampal neurons after an oxidative insult (Farinazzo et al., 2015). Moreover, exosomes derived from BM-MSC have been used to improve recovery and neuroregeneration after stroke and traumatic brain injury (Xin et al., 2013; Zhang et al., 2015).

We recently demonstrated that exosomes isolated from ASC exert a neuroprotective effect in an *in vitro* model of ALS (Bonafede et al., 2016). In this study, we used the motoneuron-like cell line NSC-34 transiently and stable transfected with different SOD1 point mutations to mimic the behavior of ALS motoneurons, and H_2_O_2_ as pathological insult. The presence of exosomes increased ALS motoneuron survival, probably counteracting the apoptosis pathway, underlining their possible therapeutic application to ALS. Another study demonstrated the possible application of ASC derived exosomes in ALS, reducing mutant SOD1 aggregation and restoring mitochondrial protein function (Lee et al., 2016a). Moreover, exosomes derived from ASC ameliorated the disease progression in an *in vitro* model of Huntington’s disease, reducing huntingtin protein aggregates and the level of apoptotic proteins (Lee et al., 2016b). Altogether, these studies indicate a protective beneficial role of exosomes in different neurodegenerative diseases.

Increasing evidence suggests that exosomes display anti-inflammatory properties, reducing the number of activated inflammatory microglial cells, supporting oligodendrocytes and protecting neurons (Zhuang et al., 2011; Pusic et al., 2014). Moreover, exosomes injected intranasally are transported rapidly to the brain, where they can be taken up by microglial cells (Zhuang et al., 2011). Since, as stated above, neuroinflammation is a common feature of neurodegenerative diseases, the suppression of the neuroinflammatory response and the possibility to reach easily the CNS are of great importance for the treatment of these pathologies.

**Figure 2:**
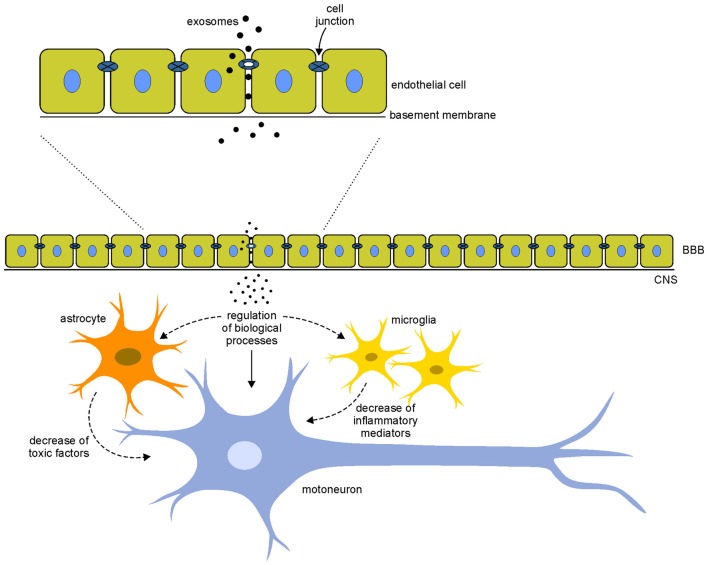
**Hypothetical mechanisms of action of exosomes.** Exosomes interact with the endothelial cells of the blood-brain barrier (BBB) modifying the integrity of cell junctions and increasing the permeability between cells. This mechanism allows a massive entry of vesicles in the central nervous system (CNS). Once in the CNS, exosomes could interact directly on motoneurons (arrows) modulating different biological processes (such as apoptosis, cell proliferation, gene expression and oxidative stress) or indirectly modifying the local motoneuron environment, acting on glial cells that decrease the release of toxic factor and inflammatory mediators (dotted arrows). These direct and indirect mechanisms of action of exosomes could counteract the pathological mechanisms involved in the disease.

The increasing attention to the use of exosomes for the treatment of neurodegenerative diseases derives from the capability of exosomes to cross the BBB, the physiological barrier that separates the brain parenchyma from circulating blood in the CNS. As mentioned before, current treatments for neurodegenerative diseases are often symptomatic and do not halt the degenerative processes; the main problem for the cure of these pathologies is the inability of cells and drugs to cross the BBB. Recent studies have reported that exosomes, unlike microvesicles, can cross the BBB: the hypothesis is that exosomes are internalized by endothelial cells and, through the release of specific cargo, they affect the integrity of cell junctions increasing permeability between cells and allowing a massive entry of vesicles in the CNS (Zhou et al., 2014; Tominaga et al., 2015; Jarmalavičiūtė and Pivoriūnas, 2016), where they could exert neuroprotective actions (Figure 2).

In this regards, we recently set up a protocol to label exosomes with superparamagnetic iron oxide nanoparticles, and the use of labeled exosomes, which allows their detection by magnetic resonance imaging, could be useful to verify this hypothesis (Busato et al., 2016).

## Conclusions and Future Perspectives

The scientific interest on MSC as potential therapeutic approach in neurodegenerative diseases is due to their ability to migrate to damaged tissues, differentiate and contribute to reparative processes (Faravelli et al., 2014). Besides these characteristics, ASC are also easily available and can be used for autologous transplantation, avoiding possible cell rejection (Marconi et al., 2013). These properties make ASC a candidate for cell therapy based on cell replacement and cell differentiation. These features have led different research groups to use ASC as possible therapy for ALS. In a study on the effects of ASC systemic administration in a murine model of ALS we have demonstrated that the treatment delayed signs of motor deterioration although a limited amount of ASC in the site of injury was detected (Marconi et al., 2013). Since the beneficial effects of ASC could be due to paracrine activity rather than to their engraftment, the scientific community has shifted the focus from cells to EVs, which, thanks to their content, can recapitulate the ASC effects and could potentially provide the basis for a non-cell-based therapy for the treatment of ALS. This innovative therapy would be easy to transfer to ALS patients since the EVs would be obtained from autologous ASC and would preserve the immune properties of their origins, avoiding immunogenic reaction.

In view of a possible application of EVs to ALS, a disease in which the BBB is not disrupted, the vesicles need to cross the BBB to reach the site of injury and, among EVs, exosomes have the required characteristics. To test the effects of exosomes in ALS, we have recently demonstrated that the administration of exosomes in an *in vitro* model of ALS exerts a neuroprotective effect following an oxidative insult, supporting the idea that exosomes can recapitulate and ameliorate the neuroprotective effect of stem cell therapy (Bonafede et al., 2016).

The evaluation of the exosomes content, in terms of proteins, mRNAs and miRNAs, and their correlation with a specific cellular pathway involved in ALS, could pave the way to explain their mechanism of action. Moreover, the understanding of the involved pathways could lead to the generation of engineered cells which release exosomes enriched with specific molecules. This could lead to a targeted exosome therapy in ALS.

## Author Contributions

RB and RM designed and wrote the review.

## Conflict of Interest Statement

The authors declare that the research was conducted in the absence of any commercial or financial relationships that could be construed as a potential conflict of interest.

## Footnotes

^1^http://www.exocarta.org
